# Supplementary material for: How Distressed Are Adolescent Students? A Mix-Method Study on High School Students in Northern Italy, Two Years after the Beginning of the COVID-19 Pandemic
Source: Behav Sci (Basel). 2024 Sep 3;14(9):775. doi: 10.3390/bs14090775 (PMC11428422; doi:10.3390/bs14090775)
Supplement: Supplementary file 1 [file behavsci-14-00775-s001.zip › behavsci-3136015-supplementary.pdf]

Supplementary Materials

Table S1. The Table shows the word forms included in the WordCloud, together with their overall frequencies in the corpus. Wordforms belonging to the same lemma are grouped together.

Word form	Translation into English	Frequency
Scuola	School	309
Non	Not	220
Studio	Study	87
Fatto	Fact/Done**	63
Ansia	Anxiety	50
Verifiche	Written tests	58
Amici	Friends	57
Stress	Distress	48
Fare<br>Faccio	To do<br>(I) do*	47<br>12
Persone	People	45
Cose	Things	43
Situazione<br>Situazioni	Situation<br>Situations	43<br>22
Problemi	Problems	40
Famiglia	Family	39
Interrogazioni	Oral tests	38
Tempo	Time	32
Pandemia	Pandemic	31
Pressione	Pressure	31
Causa	Cause	27
Genitori	Parents	27
Vita	Life	27
Casa	Home/House**	26
Stare<br>Sto<br>Sta	To stay/be/feel**<br>I stay/am/feel<br>(He, she, it) stays/is/feels*	26<br>15<br>8
Tutto	Everything	26
Sento<br>Sentirmi	(I) feel*	25<br>9
Covid	Covid	23
Penso	(I) think*	21
Spesso	Often	21
Troppe	Too many (f)***	20
Male	Bad(ly)**	19
Paura	Fear (n)***	19
Riuscire	Manage/succeed**	18
Generale	General	17
Personali	Personal (m, pl)***	17
Scolastico<br>Scolastica<br>Scolastici	School (adj, m, s)***<br>School (adj, f, s)<br>School (adj, m, pl)	17<br>14<br>12
Compiti	Homework	16
Familiari<br>Famigliare	Family (adj, m, pl)/Familiar (pl)**<br>Family (adj, m, s) (n, s)***	16<br>4

Sempre	Always	16
Solo	Alone (m)***	16
Sola	Alone (f)	10
Professori	Professors/Teachers**	15
Sport	Sport	15
Difficoltà	Difficulties	14
Solitudine	Loneliness	14
Studiare	To study	14
Volte	Times	14
Impegni	Commitments	13
Lavoro	Work/Job**	13
Poi	Then	13
Stressata	Stressed (f)***	13
Voti	Marks/Grades**	13
Futuro	Future	12
Periodo	Period	12
Principalmente	Mainly	12
Relazioni	Relations	12
Sentire	To feel	12
Altri	Other (adj or n, m, pl)***	
Altre	Other (adj or n, f, pl)***	11
Parte	Part	11
Poter	To be able to	11
Tutti	Everyone	11
Carico	Load	10
Classe	Class/classroom**	10
Così	So	10
Dover	To have to	10
Mancanza	Lack	10
Rapporti	Relationships	10
Soprattutto	Mainly	10
Uscire	To go out	10
Via	(A)Way**	10
Anno	Year	9
Credo	(I) believe*	9
Costante	Constant	9
Fatica	Effort	9
Momenti	Moments	9
Ore	Hours	9
Peso	Weight	9
Sensazione	Feeling	9
Vedere	To see	9
Abbastanza	Enough	8
Ansiosa	Anxious (f)***	8
Cambiamento	Change (n)***	8
Corpo	Body	8
Dato	Datum/Given**	8
Fine	End	8
Giorno	Day	8
Giorni	Days	8
Motivi	Reasons	8

Sociale	Social	8
Stanchezza	Tiredness	8
Trimestre	Trimester	8

* Note that in Italian it is possible to omit the subject, as the form of the verb changes depending on the person. Therefore, in the English translation, the subject is indicated in brackets. **Multiple translations are provided here in the case of polysemous words used with various meanings in the corpus, and/or in the case of words whose usage in the corpus could have different possible translations into English. ***Grammatical labels are here given when necessary: m = masculine, f = feminine, s = singular, pl = plural, n = noun.

Table S2. The Table shows the original texts of the answers cited in Section 3.1.3. of the paper.

Category	Example
School	La scuola mi fa sentire ansiosa. mi sento come se non riuscissi a far fronte a tutta la pressione a cui vengo sottoposta da tutte le cose che devo fare e dalle aspettative.
	Penso che le cause principali siano state:l attività scolastica e le pressioni messe da miei genitori derivate da essa.
	Accumulo di verifiche/interrogazioni dato dalla fine del trimestre. Ho sentito la pressione data dal fatto che, andando bene a scuola, non dovevo deludere le aspettative.
	Il grande carico di compiti e la mancata comprensione dei professori del fatto che usciamo da un periodo difficile. Aumentano i compiti e puniscono più spesso, sono più frustrati di prima!!!
	La scuola mi ha fatto sentire molto stressato nell'ultimo periodo a causa delle verifiche e di certi professori che se ne fregano dei propri studenti.
	A causa del covid, le elezione in dad, lo studio è aumentato notevolmente. Quindi mi ritrovavo a fine giornata stanca a causa del troppo studio e compiti. non avevo più tempo per me stessa, per divertirmi ecc… Stavo ore e ore davanti al computer tutti i giorni a tutte le ore.
	Il ritorno a scuola in presenza mi ha turbata molto, non riesco a stare dietro alle lezioni, concentrarmi e studiare
	io penso la scuola mia abbia depresso e cambiato in male perché non ho più la ispirazione di fare nulla
	Troppo studio, l accavallarsi di verifiche e interrogazioni. La mia mente era distrutta è il mio corpo ne ha risentito
	Ho avuto difficoltà con la scuola: soffro di attacchi di panico e ansia e quando mi trovo con troppe cose da fare e vado in ansia. In 20 giorni ho tentato due volte di morire prendendo una quantità esagerata di pastiglie, finendo anche in ospedale.
	Mi sentivo troppo caricata dalla scuola e piena di responsabilità, causata anche dal fatto che sono all’ultimo anno di liceo.
Interpersonal relationship	Litigi con il mio gruppo di compagne di scuola, mi hanno “insultatx”, esclusx, isolatx, e messo la maggior parte della classe contro.
	Non sentirmi capita, essere meno energica, sentirmi un vuoto dentro provocato da delusioni causate da persone un tempo importanti nella mia vita e di conseguenza non riuscire a fidarmi delle persone.
	le cause sono genitori oppressivi che non accetterebbero la mia omosessualità essendo che già non mi accettano
	Mi sento ripetere le stesse cose da mesi, è stancante. I miei genitori dovrebbero anche parlare di qualcos’altro oltre lo studio, altrimenti la mia autostima non fa che abbassarsi.
	La mancanza di relazioni ha causato una perdita nella capacità di relazionarmi, provocandomi una sensazione di inadeguatezza quando sono in gruppo

Negative emotional states	Ci sono tante cose che mi fanno senitre in ansia, sono costantemente in tensione.<br>Depressione e ansia che hanno creati situazioni complicate come l'autolesionismo.<br>Ultimamente sto perdendo la voglia di uscire, preferisco rimanere nella mia zona di comfort, nella mia camera. non trovo più stimoli per uscire e non mi interessa prendermi cura di me stessa. Dopo aver smesso di praticare il mio sport, il nuoto, salto molto spesso i pasti anche se ho fame. Mi sento molto spesso triste e avvilita e le mie ore di sonno stanno calando molto rapidamente. Ho anche notato che sono più sensibile e irritabile da quando è iniziata la pandemia. Nel periodo iniziale di questa tragedia mi è purtroppo capitato di cadere i una situazione di solitudine dove ho trovato sollievo nell'autolesionismo, che ho, tristemente, ripreso da poco.<br>La mia mente in questo lungo periodo che coincide con l'inizio della pandemia fino ad oggi ha subito un percorso di cambiamento radicale e ad oggi sicuramene la situazione è molto negativa rispetto a prima, per via della nascita di innumerevoli situazioni di sconforto, attacchi di panico e momenti di pressione o depressione, che hanno influito molto a livello emotivo. Di sicuro tutto ciò è legato anche alla nascita di problemi e comportamenti ossessivi nei confronti del mio corpo, che è totalmente cambiato per via di una perdita di più di 30kg, sia nei confronti dell'allenamento e soprattutto dell'alimentazione, che oggigiorno mi hanno portato a dover seguire un percorso assistito di tipo psicologico e nutrizionale.<br>Ho iniziato a pensare alla vita, come potrebbe finire in ogni momento. Ho iniziato a pensare di non essere abbastanza per gli altri (ma lo penso io). Sono anche spaventata da me stessa perché talvolta penso all'opzione del suicidio e mi terrorizza il solo pensiero di poter fare qualcosa che può far soffrire gli altri.
Covid-19 pandemic	Il fatto di essere chiusa in casa con i miei genitori e mia sorella e non vedere i miei amici da molto tempo.<br>Penso il fattore di stare chiuso in casa e non uscire mai, non avere più il contatto con le persone e non praticare lo sport che amavo.<br>Durante la pandemia ho avuto molto tempo per riflettere e passare tempo con la mia famiglia molto tossica e questo mi ha fatto molto male ho avuto svariati pensieri di farla finita, ero e sono distrutta mentalmente ma nessuno se ne accorge
Overall negative, but otherwise unspecified, emotional state	Un'insoddisfazione generale riguardo diversi aspetti dell vita in cui sono in difficoltà che non mi permettono di concentrarmi anche su altri aspetti riguardo ai quali dovrei essere più sereno.<br>bho non lo so..non saprei non c'è un motivo valido..<br>Ansia inspiegabile, per non so che cosa!
Feelings of isolation and loneliness	Il fatto che mi sentissi tremendamente solo, cercavo aiuto ma nessuno riusciva a darmelo, ne in famiglia ne al di fuori.
Other	Probabilmente questa difficile situazione in cui ci troviamo, i dispositivi elettronici che stanno creando dipendenza. Questo, nel mio caso influisce molto sul sonno, è difficile addormentarsi e spesso mi sveglio nel bel mezzo della notte.<br>Perdo troppo tempo con il cellulare e non riesco a limitarne l'utilizzo, ho difficoltà ad organizzarmi con lo studio sia per vie del cellulare che per la difficoltà a concentrarmi, sono consapevole di buttare via il mio tempo ma non riesco a cambiare questa situazione.<br>Problemi con il mio corpo e il cibo.
